# Functional screening system for yeast-secreted peptides acting on G-protein coupled receptors

**DOI:** 10.1186/s13568-015-0113-8

**Published:** 2015-05-13

**Authors:** Tomohiro Shigemori, Kouichi Kuroda, Mitsuyoshi Ueda

**Affiliations:** Division of Applied Life Sciences, Graduate School of Agriculture, Kyoto University, Sakyo-ku, Kyoto, 606-8502 Japan

**Keywords:** Functional screening system, G-protein coupled receptors, Peptides, Yeast, Glucagon-like peptide-1

## Abstract

We established a novel functional screening system for peptides acting on G-protein coupled receptors (GPCRs). Peptides are a promising drug scaffold because of their intermediate molecular size between that of therapeutic small molecules and antibodies. They also offer potential advantages of targeting not only membrane proteins but also intracellular protein–protein interactions. Phage display technology has been used for exploring novel peptides acting on GPCRs, but it is unclear whether the identified peptides functionally modulate targets because the technology selects peptides based on binding ability but not functional activity to targets. In a novel screening system that we established, yeast cells were utilized as a peptide producer while mammalian cells stably producing the receptor for glucagon-like peptide 1 (GLP1R) were used as a biosensor for receptor activation. Three kinds of GLP1R agonists secreted by yeasts were successfully detected for their functional activities without any purification and condensation of those peptides. By applying the functional screening system, we were able to identify GLP1R agonist-secreting yeasts based on GLP1R activation from the cell mixture containing a number of background yeasts that produced non-active control peptides. Further applications of this system would include not only activity evaluation of bioactive peptides without chemical synthesis but also discovery of novel peptides activating druggable GPCRs.

## Introduction

G-protein coupled receptors (GPCRs) are one of critical eukaryotic signal transduction gatekeepers and represent the largest protein family in the human proteome with more than 800 members. They share a common architecture of seven transmembrane helices, and can be classified into five major classes of sequence similarities (Jacoby et al. 2006): rhodopsin receptor family (class A), secretin-like receptor family (class B), glutamate receptor family (class C), frizzled/taste 2 receptor family, and adhesion receptor family. GPCRs recognize a variety of extracellular stimuli, including photons, ions, small molecules, peptides, and proteins; they transmit the resulting extracellular signals across the membrane to elicit intracellular responses. Consequently, GPCRs are involved in physiological and pathophysiological changes in blood pressure, blood sugar, pain, allergies, and so on. Therefore, pharmacological activation or suppression of GPCRs has been effective means to treat various diseases related to GPCR dysregulation.

Peptides are involved in a variety of physiological and pathological processes, and play very important roles in modulating various cell functions such as the absorption of blood glucose into the body through the promotion of insulin secretion in pancreatic β-cells by glucagon-like peptide-1 (GLP1), a peptide hormone that are postprandially secreted in intestinal L-cells and activates one of class B GPCRs, GLP1 receptor (GLP1R) (Baggio and Drucker 2007). Because of their intermediate molecular size ranging from 0.5 to 5 kDa which is between that of small-molecule drugs and therapeutic monoclonal antibodies, peptides potentially have advantages of easy drug design, high safety, accessibility for protein–protein interactions, targeting of intracellular molecules, and low production cost (Craik et al. 2013).

Phage display is the first innovative technology established by Smith (1985) that allows researchers to prepare and screen a large polypeptide library. However, this methodology is not effective at discovering functional peptides such as activators for GPCRs. This is due to the nature of phage display, whose peptides are screened based only on binding ability to targets, resulting frequently in simple binding peptides without any bioactivity (Chen et al. 2007). Furthermore, the identified peptides on the phage are never a functional, soluble form, potentially leading to dissociation in activity between peptides tied up on the phage as a fusion protein and the secretion form of peptides.

The yeast *Saccharomyces cerevisiae* is suitable as host for production of peptide library; this is due to their abundance in gene manipulation tools, fast growth in a low-cost medium, and protein folding and secretory machinery homologous to that of mammalian cells (Idiris et al. 2010). In this study, we report a novel functional screening method for bioactive peptides acting on GPCRs, which integrated a yeast secretion system and a functional detection system using GPCR-producing mammalian cells. GLP1R produced on mammalian cells was successfully activated by various GLP1R agonistic peptides that were secreted from yeast. We were also able to identify GLP1R agonist-secreting yeasts based on GLP1R activation from the cell mixture containing a number of background yeasts, which produced non-active control peptides, suggesting the effectiveness of our functional screening system to discover novel peptide-based drugs acting on GPCRs.

## Materials and methods

### Strains and media

*Esherichia coli* DH5α [*F*^−^*, ΔlacU169* (*φ80lacZΔM15*)*, hsdR17* (*r*_*K*_^−^*, m*_*K*_^+^)*, recA1, endA1, deoR, thi*-*1, supE44, gyrA96, relA1, λ*^−^] (Toyobo, Osaka, Japan) was used as a host for DNA manipulation. *E. coli* transformants were grown in Luria–Bertani (LB) medium [1% (*w*/*v*) tryptone, 0.5% (*w*/*v*) yeast extract, 1% (*w*/*v*) sodium chloride, and 2% (*w*/*v*) agarose] containing 100 µg/mL ampicillin or kanamycin depending on the plasmids introduced.

*Saccharomyces cerevisiae* BY4742 (*MATα*, *his3Δ1*, *leu2Δ0*, *lys2Δ0*, *ura3Δ0*; EUROSCARF, Frankfurt, Germany) was used to construct the yeasts secreting GLP1R agonists, including GLP1, S^2^-GLP1 substituted with serine at the position 2 of GLP1, and exendin-4 (Ex4), a naturally occurring peptide found in the saliva of the Gila monster (Furman 2012). Yeast transformants were selected on synthetic dextrose (SDC) solid medium [0.67% (*w*/*v*) yeast nitrogen base without amino acids, 2% (*w*/*v*) glucose, 1% (*w*/*v*) casamino acids, 0.002% (*w*/*v*) adenine, 0.002% (*w*/*v*) l-tryptophan, and 2% (*w*/*v*) agar], and then, the resultant colonies were cultivated in 6-well plate (353046; Thermo Fisher Scientific, Waltham, MA, USA) or 96-well plate (353072; Thermo Fisher Scientific) containing a liquid SDC medium or Dulbecco’s modified Eagle Medium (DMEM) (Nacalai Tesque, Kyoto, Japan) at 30°C.

Chinese hamster ovary (CHO) cells (85050302; European Collection of Cell Cultures, Salisbury, UK) were used as a host cell stably producing human GLP1R and cultivated in Ham-F12 (Sigma-Aldrich, St. Louis, MO, USA) containing 10% FBS (Thermo Fisher Scientific) and 400 μg/mL G418 (Nacalai Tesque).

### Construction of peptide-secreting yeast

All the primers used in plasmid construction are listed in Table 1. For peptide secretion in yeast, pULS harboring the engineered secretion signal of yeast α-factor, appS4 (Rakestraw et al. 2009), in the downstream of GAPDH promoter, was constructed as follows. The DNA fragment encoding the appS4 (FASMAC, Kanagawa, Japan) in pUC19 was amplified using primers 1 and 2, and was inserted into pULI1 (Miura et al. 2012), which was digested with *EcoR*I and *Xba*I by using In-Fusion (Clontech Laboratories, Inc., Mountain View, CA, USA) to obtain pULS. The DNA fragments encoding GLP1, S^2^-GLP1 and Ex4, with or without a FLAG-encoding sequence at the 3′ terminus, were double-stranded from oligonucleotides with the mutual complementary region using DNA polymerase KOD-FX-Neo (Toyobo). The double-stranded DNAs encoding those GLP1 analogues were introduced into the multiple cloning sites of pULS by In-Fusion (Clontech) and named pULS-GLP1, -GLP1FLAG, -S^2^-GLP1, -S^2^-GLP1FLAG, -Ex4 and -Ex4FLAG, respectively. Yeasts were transformed with those plasmids using Frozen-EZ Yeast Transformation-II kit (Zymo Research, Orange, CA, USA), resulting in GLP1-yeast, GLP1F-yeast, S^2^-GLP1-yeast, S^2^-GLP1F-yeast, Ex4-yeast, and Ex4F-yeast, respectively. Yeast transformed with pULS (Ctrl-yeast) was used as control.

**Table 1:** Primers used in this study

	Sequence
Primer 1	5′-AAACACACATAAACACCCGGGATG-3′
Primer 2	5′-CAGTCTAGAGGATCCGAATTCTCTTTTATCCAAAGATACCCCTTCTTC-3′
Primer 3	5′-CGAGCTCGGATCGATCGCCACCATGGCCGGCGCCC-3′
Primer 4	5′-TATCTATGCGGCCGCTCAGCTGCAGGAGGCCTG-3′
Primer 5	5′-GAAGAAGGGGTATCTTTGGATAAAAG-3′
Primer 6	5′-CTTGTCATCGTCATCCTTGTAATC-3′

Underlines indicate homologous region to the corresponding plasmids.

### Construction of human GLP1R-producing CHO

Human GLP1R gene was PCR-amplified from the human brain cDNA library (BioChain, Newark, CA, USA) using DNA polymerase KOD-Plus-Neo (Toyobo) with primers 3 and 4 (Table 1). The DNA fragment coding human GLP1R was inserted into pIRES (Clontech) digested with *Eco*RV and *Bam*HI by using In-Fusion (Clontech), resulting in pIRES-hGLP1R. CHO cells were transfected with pIRES-hGLP1R using Xfect (Clontech), and then, selected with G418 for about 2 weeks to construct a stable cell line producing hGLP1R. Single cell cloning of the resistant cells was conducted by limiting dilution, resulting in GLP1R-CHO.

### GLP1R activation assay using GLP1R-CHO

GLP1R-CHO was seeded onto a 96-well plate at 5 × 10^4^ cells and cultured at 37°C for 24 h. After the cells were washed with HANKS buffer (Thermo Fisher Scientific), synthetic GLP1R agonists (GLP1 and Ex4; Peptide Institute, Osaka, Japan. S^2^-GLP1; Bachem, Bubendorf, Switzerland) or culture supernatants of GLP1R agonists-secreting yeast were added and incubated at 37°C for 45 min. Then, the cells were lysed with Assay/Lysis buffer (Thermo Fisher Scientific) and the level of cyclic AMP in the cell lysate was determined by using the cAMP-screen^®^ assay (Thermo Fisher Scientific) according to the manufacturer’s instructions.

### Model screening of Ex4-secreting yeast

Ten yeast cells comprised of Ctrl-yeast and Ex4-yeast in the theoretical ratio of 9:1 were seeded into 16 wells in 96-well plate containing 300 µL of SDC medium and grown for 48 h. Then, the medium was exchanged into 250 µL of DMEM and yeasts were additionally cultivated for 12 h at 30°C. After that, the supernatant was subjected to the GLP1R activation assay mentioned above. Yeasts included in three wells showing or not showing activity were seeded on SDC solid medium to form single colonies. Then, 48 colonies were subjected to colony-direct PCR with primers 5 and 6 (Table 1), and the resultant PCR products were analyzed by agarose gel electrophoresis to identify yeasts with the Ex4 gene.

## Results

### Construction of stable CHO cells producing human GLP1R

**Figure 1:**
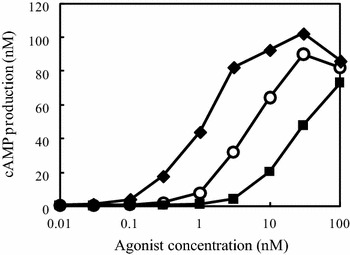
Dose–response curve of GLP1R activation in GLP1R-CHO upon treatment of synthetic GLP1R agonists. GLP1R-CHO cells were exposed to three kinds of GLP1R agonists, GLP1 (*white circle*), S^2^-GLP1 (*black square*) and Ex4 (*black diamond*), at increasing concentrations. After 45 min incubation, cells were lysed and cAMP production was determined by ELISA as indicated in “Materials and methods”.

CHO cells producing human GLP1R (GLP1R-CHO) were constructed to detect functional activity of synthetic- or yeast-secreted GLP1R agonists. CHO cells were transfected with pIRES-hGLP1R by lipofection and selected using the neomycin resistance gene on the pIRES vector. The established cells showed cAMP production upon GLP1R activation by three kinds of synthetic GLP1R agonists, GLP1, S^2^-GLP1, and Ex4 in a dose-dependent manner, with EC_50_ value of 5.8, 21.5 and 1.4 nM, respectively (Figure 1; Table 2). Therefore, functional human GLP1R was successfully produced in CHO cells.

**Table 2:** Amino acid sequences of GLP1R agonists used in this study

Agonists	Amino acid sequence	EC_50_ (nM)
GLP1	HAEGTFTSDVSSYLEGQAAKEFIAWLVKGR	5.8
S^2^-GLP1	HSEGTFTSDVSSYLEGQAAKEFIAWLVKGR	22
Exendin4	HGEGTFTSDLSKQMEEEAVRLFIEWLKNGGPSSGAPPPS	1.4

Underlines indicate amino acids different from GLP1.

### Medium optimization for GLP1R activation by yeast-secreted peptides and establishment of assay system

**Figure 2:**
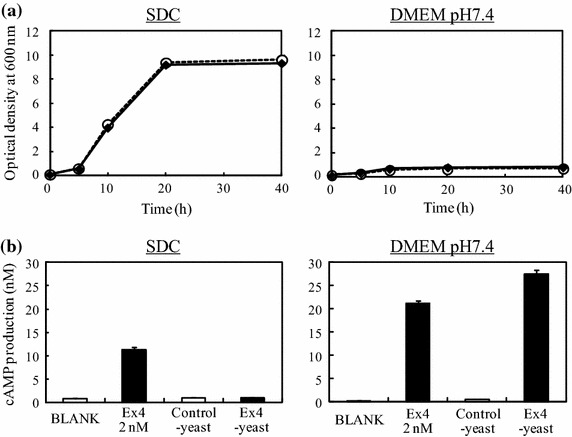
Medium optimization for GLP1R activation assay of yeast-secreted peptides. Yeasts were grown in SDC and DMEM on 6-well plate for 40 h, and then, growth rate (**a**) and GLP1R activation potential of their culture supernatant (**b**) were evaluated by measurement of absorbance at 600 nm and ELISA for cAMP, respectively. For growth curve, *white circle* indicates Ctrl-yeast and *black diamond* shows Ex4-yeast. The data represent the average ± SEM of three independent experiments.

We first tested which medium was suitable for yeast growth and evaluation of GLP1R activation in GLP1R-CHO in 6-well plates. Ctrl-yeast and Ex4-yeast were inoculated in a 6-well plate containing SDC medium or DMEM at the initial optical density (OD) which was 600 nm of 0.1 and incubated for 40 h. Then, the growth rates of yeasts and the GLP1R activation in GLP1R-CHO by culture supernatant containing yeast-secreted peptides were evaluated (Figure 2). As a result, SDC gave a higher growth reaching stationary phase at 20 h, showing about 90-fold expansion. On the other hand, yeasts cultivated in DMEM showed much lower growth, reaching only the maximal OD_600_ of 0.75 at 20 h (Figure 2a). For GLP1R activation potency after the 40-h cultivation, the culture supernatant of Ex4-yeast grown in SDC showed no activity, although exogenously added Ex4 activated GLP1R by 16-fold, compared with the control. In contrast, Ex4-yeast grown in DMEM strongly induced GLP1R activation by 50-fold compared to the Ctrl-yeast (Figure 2b). These results indicate that SDC medium is suitable for yeast proliferation, while DMEM is excellent for GLP1R activation.

**Figure 3:**
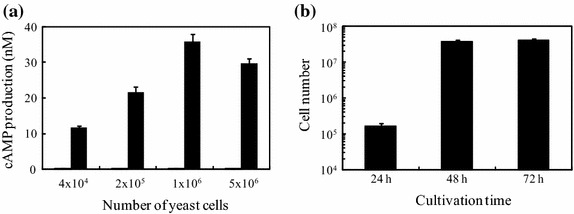
Scale-down assay for GLP1R activation by yeast-secreted peptides. **a** Indicated number of yeast cells, Ctrl-yeast (*left bar*) or Ex4-yeast (*right bar*), were seeded in a 96-well plate containing DMEM and incubated for 12 h. Then, GLP1R activation assay was conducted to find the yeast cell number providing wider dynamic range. The data represent the average ± SEM of three independent experiments. **b** Yeast cultivation time to achieve the target yeast cell number, 1 × 10^6^, was evaluated. Yeasts were seeded in a 96-well plate containing SDC at the initial number of 3. After 24-, 48-, and 72-h cultivations, the number of yeasts was determined using hematometer. The data represent the average ± SEM of three independent experiments.

Next, we tried to establish a GLP1R activation assay system combined with peptide-secreting yeast in a 96-well plate format. We first determined the number of Ex4-yeasts required for larger dynamic range in the GLP1R activation. Yeast cells suspended in DMEM were prepared at 4 × 10^4^ to 5 × 10^6^ cells in 96-well plates, and after a 12 h-cultivation at 30°C, a GLP1R activation assay using the culture supernatants was carried out (Figure 3a). The results showed that 4 × 10^4^ yeast cells were enough to detect GLP1R activation, showing 73-fold activation, compared with the control, and 1 × 10^6^ yeast cells provided the largest dynamic range with 224-fold activation of GLP1R. Further, we investigated the cultivation time required for the yeast cell number of 1 × 10^6^ (Figure 3b). Yeast preparations with the initial cell number of 3 were cultivated in SDC medium that showed the highest growth as in Figure 1, and then, yeast cell number was counted at 24, 48, and 72 h cultivation. As results, the cell number per initial cell number exceeded 1 × 10^6^ cells after 48 h cultivation. These results suggested that a single yeast cell can reach 1 × 10^6^ cells, showing the highest dynamic range in GLP1R activation assay when cultivated in SDC for 48 h.

### Direct functional assay of various yeast-secreted GLP1R peptide agonists

**Figure 4:**
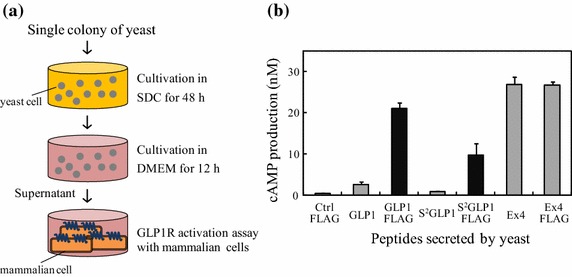
Direct activity detection of various yeast-secreted GLP1R agonists. **a** Schematic illustration for direct activity detection assay was depicted. Single colony of yeasts was inoculated in 96-well plate containing SDC and cultivated for 48 h. After removal of SDC, DMEM was added and incubated for additional 12 h. Then, the culture was subjected to GLP1R activation assay using GLP1R-CHO. **b** Seven kinds of yeasts were tested for GLP1R activation potency according to the assay flow mentioned above. The data represent the average ± SEM of three independent experiments.

Based on these optimizations, we constructed a series of workflow for direct functional assay of yeast-secreted peptides on GLP1R, composed of three steps (Figure 4a): cultivation for yeast growth, peptide secretion in yeasts, and GLP1R activation assay using GLP1R-CHO. To demonstrate the effectiveness of the direct functional assay system, we attempted to detect the GLP1R activation by native GLP1, its analogue S^2^-GLP1, and Ex4 secreted by yeast. Single colonies of the yeasts secreting GLP1, S^2^-GLP1, and Ex4 were inoculated and incubated in a 96-well plate containing SDC for 48 h, and then, the medium was exchanged into DMEM with additional incubation of 12 h, followed by the GLP1R activation assay (Figure 4b). While yeast-secreted Ex4 showed the highest activation by 59-fold, GLP1 and S^2^-GLP1 secreted by yeasts provided only 5.8 and 1.7-fold activation, respectively. Therefore, GLP1 and S^2^-GLP1 were considered insufficient given those EC_50_ values are a quarter and one sixteenth of the Ex4 values, respectively. Whereas the N-terminal two residues (His-Ala) in GLP1 are reported to be critical for its biological activity, the C-terminal part is tolerable to various modifications such as fatty acid conjugation or albumin fusion (Baggio et al. 2004; Madsen et al. 2007). Thus, we fused FLAG tag to the C-terminus of GLP1, S^2^-GLP1, and Ex4 initially for affinity purification and Western blotting, if needed. Remarkably, when we evaluated the GLP1R activation potency for such yeast-secreted GLP1R agonists with the FLAG tag, GLP1 and S^2^-GLP1 activated GLP1R about 10-times higher than those without the FLAG tag, respectively. These results demonstrated that yeast-secreted GLP1 and S^2^-GLP1 with the addition of C-terminal FLAG tag were successfully and directly detected without any purification and condensation by using the novel functional detection system established here.

### Examination of model screening using our established functional assay system

**Figure 5:**
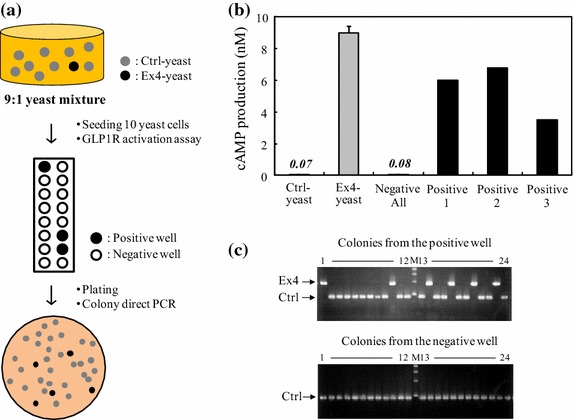
Model screening of Ex4-secreting yeast using the direct activity detection system. **a** Schematic illustration for model screening of Ex4-yeast was indicated. **b** We prepared 16 wells including 9:1 yeast mixture (10 cells in total per well) in the ratio of Ctrl-yeast to Ex4-yeast and conducted GLP1R activation assay according to the workflow indicated above. Only Ctrl-yeast or Ex4-yeast was tested as a control. **c** The existence of Ex4-yeast in the positive and negative wells was investigated by colony-direct PCR after the colony formation of yeasts from both wells (numbering from 1 to 24 means the number of colony picked up). The representative electrophoresis data are shown for colonies from a positive and a negative well, respectively. *M* molecular size marker (New England Biolabs, Ipswich, MA, USA).

Finally, we performed a model screening of yeast by applying the direct functional assay system, for example, a screening of Ex4-yeast in the co-presence of excess Ctrl-yeast. A yeast cell mixture including Ctrl-yeast and Ex4-yeast at a theoretical ratio of 9:1 was prepared, and a GLP1R activation assay was conducted according to the determined workflow (Figure 5a). As a result, we identified three positive wells showing GLP1R activation (Figure 5b). We next investigated the existence of Ex4-yeast in the positive and negative wells by colony-direct PCR after the colony formation of yeasts from both wells. The results indicated the existence of 24% of Ex4-yeast in the positive wells, whereas Ex4-yeast was not identified at all in the negative wells (Figure 5c). These results clearly suggest that our direct functional assay system established was effective in the model screening.

## Discussion

In this study, we established the novel functional screening system of yeast-secreted peptides acting on GLP1R. The system directly detected the functional activity of yeast secreted-GLP1R agonists based on activation of GLP1R produced on mammalian cells, without any purification and condensation of yeast-produced peptides. In addition, application of the system enabled identification of agonist-secreting yeast in the model screening.

Binding-based peptide screening strategy as represented by phage display has been the most common method used to discover novel peptide-ligands for certain drug targets, including GPCRs (Molek et al. 2011; Vyroubalova et al. 2006). However, with peptides discovered through this methodology, it is uncertain whether they are biologically active or not. In addition, phage-displayed peptides are distinct from functional peptides in their soluble form. These conventional challenges result in additional chemical synthesis of the identified peptides in soluble form to evaluate their true biological activity, which are time-consuming and quite expensive.

In the first optimization step using Ex4-yeasts as a model for our novel functional assay system, we remarkably found that DMEM buffered at neutral pH designed for mammalian cell culture was very suitable for yeast peptide secretion and GLP1R activation in mammalian cells, even though yeast cells could not grow in the medium. Another remarkable point in this culture system is a protection of the target peptides from degradation by yeast-derived proteases. Because heterologous proteins produced in yeasts could be degraded during several steps, including the intracellular secretory pathways and the post-secreted extracellular environment (Idiris et al. 2010), yeast-derived proteases such as Yps1p and Kex2p, which are most active at a mild acidic condition around pH 5.0, are considered accessible to heterologous proteins, especially when yeasts are incubated in SDC medium, which generally has pH 4.5–5.5 (Mizuno et al. 1989; Olsen et al. 1999). In addition, yeasts were a good tool for producing peptides because yeasts swiftly grew in SDC medium; they even in a static 96-well plate setting which would be compatible with high-throughput screening and could easily reach the targeted yeast cell number providing high dynamic range in GLP1R activation assay (Figure 2).

These findings prompted us to conduct the direct functional detection of other GLP1R agonists with weaker activity than Ex4, GLP1, and S^2^-GLP1 to be secreted by yeasts. It was difficult to detect their GLP1R activation potency at the high level as expected from the difference of the EC_50_ value of Ex4. Surprisingly, the C-terminus fusion of the FLAG tag with DYKDDDDK in GLP1 and S^2^-GLP1 much increased the GLP1R activation potency by about tenfold compared to those without the FLAG tag (Figure 4b). In spite of the fact that the precise mechanisms of the FLAG are still uncertain, the beneficial effect of the FLAG fusion was also observed in somatostatin that is endogenous circular peptide agonist acting on class A GPCR SST receptor (data not shown). Accordingly, the FLAG fusion to isolated peptides would be a promising way to increase their activity.

As shown in Figure 5, we successfully identified the wells including Ex4-yeast by evaluating the GLP1R activation potency, and importantly, the wells that did not show activity did not possess any Ex4-yeast, as confirmed by colony-direct PCR detecting Ex4 gene. This successful model screening of yeasts secreting agonists acting on GLP1R encourages us to carry out an actual screening for novel bioactive peptides through our direct functional detection system.

In conclusion, we successfully established a novel system for direct functional assay for yeast-secreted peptides on GLP1R. This system will be applied not only for biological activity assay of sequenced peptides instead of their chemical synthesis but also discovery of novel bioactive peptides.

## Abbreviations

CHO: Chinese hamster ovary
DMEM: Dulbecco’s modified Eagle medium
Ex4: exendin-4
GLP-1: glucagon-like peptide-1
GPCR: G-protein coupled receptor.
